# Serum Levels of Interleukin-6 and Titers of Antibodies against *Porphyromonas gingivalis* Could Be Potential Biomarkers for the Diagnosis of Oral Squamous Cell Carcinoma

**DOI:** 10.3390/ijms20112749

**Published:** 2019-06-04

**Authors:** Dae-Gun Park, Bok Hee Woo, Byung-Joo Lee, Sanggyeong Yoon, Youngseuk Cho, Yong-Deok Kim, Hae Ryoun Park, Jae Min Song

**Affiliations:** 1 Department of Oral Pathology, School of Dentistry, Pusan National University, Yangsan 50612, Korea; dgnprk@gmail.com (D.-G.P.); vokiwoo@pusan.ac.kr (B.H.W.); 2 Periodontal Disease Signaling Network Research Center, School of Dentistry, Pusan National University, Yangsan 50612, Korea; 3 Department of Otorhinolaryngology-Head and Neck Surgery, School of Medicine and Biomedical Research Institute, Pusan National University, Yangsan 50612, Korea; voiceleebj@gmail.com; 4 Department of Statistics, College of Natural Science, Pusan National University, Busan 46241, Korea; tkd_rud@naver.com (S.Y.); choys@pusan.ac.kr (Y.C.); 5 Department of Oral and Maxillofacial Surgery, School of Dentistry, Pusan National University, Yangsan 50612, Korea; ydkimdds@pusan.ac.kr; 6 Dental and Life Science Institute, School of Dentistry, Pusan National University, Yangsan 50612, Korea

**Keywords:** *Porphyromonas gingivalis*, *Fusobacterium nucleatum*, immunoglobulin G, interleukin-6, oral squamous cell carcinoma, serum biomarkers

## Abstract

It has been suggested that *Porphyromonas gingivalis* (*P. gingivalis*), a keystone pathogen in chronic periodontitis, is associated with a variety of cancers, including oral cancer. Recently, studies have shown the effects of persistent exposure to *P. gingivalis* on the promotion of tumorigenic properties of oral epithelial cells, suggesting that chronic *P. gingivalis* infection is a potential risk factor for oral cancer. On the other hand, *Fusobacterium nucleatum* (*F. nucleatum*), one of the major periodontal pathogens, has emerged as an important factor in the colon cancer progression. Here, we investigated the diagnostic potential of serum immunoglobulin G antibody against periodontal pathogens, *P. gingivalis and F. nucleatum*, and serum IL-6 for oral squamous cell carcinoma (OSCC). An enzyme-linked immunosorbent assay (ELISA) was used to determine and compare the serum levels of interleukin 6 (IL-6), *F. nucleatum* IgG, and *P. gingivalis* IgG in 62 OSCC patients with 46 healthy controls. The serum levels of *P. gingivalis* IgG and IL-6 were higher in OSCC patients than in non-OSCC controls, and the difference was statistically significant. In addition, a high serum level of IL-6 was associated with a worse prognosis in OSCC patients. Thus, *P. gingivalis* IgG and IL-6 could be utilized as potential serum biomarkers for the diagnosis of OSCC, and the serum level of IL-6 contributes to improved prognostic performance.

## 1. Introduction

One of the most serious complications in surgical treatment for oral cancer is functional and cosmetic damage from surgery [1,2]. To lessen the damage, numerous therapeutic modalities such as radiotherapy, chemotherapy, and immunotherapy that could replace surgical treatment have been continuously developed, but the modalities have been successful for other types of cancer, such as leukemia, lymphoma, and breast cancer, rather than oral cancer [3,4,5,6,7,8,9]. These therapies cannot be applied for oral cancer as a primary solution and can only be used as an adjuvant therapy [10]. Due to this limitation, minimizing the need for surgery through the early detection of oral cancer is imperative to diminish undesirable outcomes. Oral squamous cell carcinoma (OSCC), the most common histopathological subtype of oral cancer, is mainly diagnosed by excising suspicious lesions and observing the morphologic features [11]. Early diagnosis of OSCC prior to grossly identifiable lesions may help to decrease the side effects of surgical treatment, and this can be achieved through a thorough understanding of the etiology and contributing factors of OSCC.

Among numerous causative and predisposing factors in cancer, chronic inflammation has been identified as one of the most important contributing factors in cancer progression. For example, patients with a history of hepatitis C virus infection are known to have a high probability of developing hepatocellular carcinoma [12]. In addition, ulcerative colitis has recently been reported to be intimately associated with progression to colon cancer [13]. These correlations have been proven through both epidemiological and in vitro and in vivo studies [14,15,16,17]. Some epidemiological studies have also suggested a positive correlation between various types of cancers, including oral cancer and periodontitis, which is the most common chronic inflammatory disease of the oral cavity [18,19,20,21]. A few experimental studies have been performed to support the results of epidemiological studies showing that chronic periodontitis is one of the key factors in a variety of cancers, especially oral cancer [22,23]. To mimic chronic periodontitis, the studies used *P. gingivalis*, a major pathogen in chronic periodontitis. *P. gingivalis* has been suggested to be a keystone bacterium in periodontitis-associated diseases, such as atherosclerosis, premature birth, and cancers [24,25,26]. Our recent study revealed that OSCC cells that had been chronically infected with *P. gingivalis* exhibited increased aggressiveness compared to noninfected cells [22]. We also observed that *P. gingivalis* treatment provided OSCC cells with resistance to paclitaxel [27]. In addition, *F. nucleatum* is also known to play an important role in the progression of periodontal disease by acting as a bridge to aggregate various facultative and obligate anaerobic periodontopathic bacteria and thus stabilize the survival of strict anaerobes, such as *P. gingivalis* [28]. As titers of antibodies specific for *P. gingivalis* and *F. nucleatum* may reflect the severity and history of periodontitis, we sought to identify the correlation between periodontitis and OSCC by comparing the immunoglobulin G value for these pathogens with clinical significance for the diagnosis and postoperative prognosis of OSCC. To further confirm the correlation between chronic periodontitis and oral cancer, we also investigated the serum levels of interleukin-6 (IL-6), which is the most representative inflammatory marker. In addition, this study could be further analyzed to evaluate the possibility of these serum markers as measures for screening, early diagnosis, and clinical management of oral cancer.

## 2. Results

### 2.1. P. gingivalis Is More Closely Associated with OSCC than F. nucleatum

The serum values of IgG against *P. gingivalis* or *F. nucleatum* and the serum levels of IL-6 were analyzed according to the demographic characteristics of OSCC patients by testing the mean differences, and the results are summarized in Table 1, Table 2 and Table 3. There were no significant differences in serum IL-6 and the titers of antibodies against *P. gingivalis* or *F. nucleatum* based on age, sex, smoking status, or alcohol use. The associations of the serum levels of IL-6, *P. gingivalis* IgG, and *F. nucleatum* IgG with clinical stage and pathological features, including lymph node metastasis, were also assessed by testing the mean differences, and this analysis also showed negative results. The serum values of *P. gingivalis* IgG were higher in histopathological grade IV and stage IV cancers than in cancers of lower grades and stages, but this difference was statistically insignificant.

**Figure 1:**
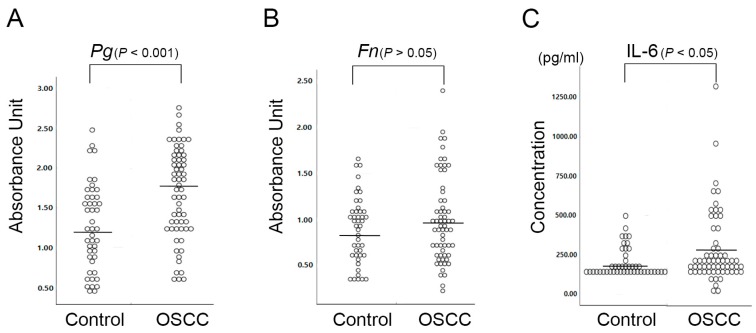
Serum antibodies against *P. gingivalis* and *F. nucleatum* and serum IL-6 levels in OSCC patients were compared with those in healthy controls using an enzyme-linked immunosorbent assay (ELISA). The serum levels in healthy controls (*n* = 46) and OSCC patients (*n* = 62) were (**A**) *P. gingivalis* IgG, 1.25 ± 0.54 and 1.69 ± 0.57, (**B**) *F. nucleatum* IgG, 0.88 ± 0.36 and 1.00 ± 0.46, (**C**) IL-6, 199.51 ± 89.38 (pg/mL) and 274.93 ± 228.57 (pg/mL), respectively.

To determine whether periodontitis and/or inflammatory status are linked to OSCC, the serum levels of antibodies against periodontal pathogens and the levels of IL-6 in OSCC patients were compared with those in healthy controls (Figure 1). The serum levels of *P. gingivalis* IgG were significantly higher in OSCC patients than in healthy controls (*p* < 0.001, multivariate analysis). The mean serum level of *F. nucleatum* IgG in OSCC patients was also higher than that in healthy controls, although the difference was statistically insignificant (*p* = 0.196, multivariate analysis). In addition, the correlation between inflammation and OSCC and the role of IL-6 was investigated. The concentrations of IL-6 were significantly elevated in OSCC patients compared to healthy controls (*p* = 0.046, Mann-Whitney U test).

**Figure 2:**
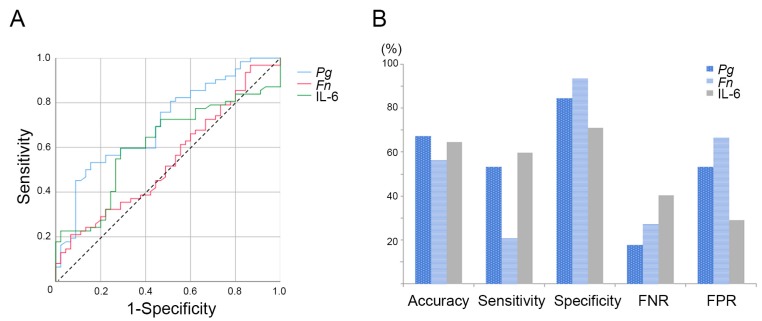
Receiver operating characteristic (ROC) curves (**A**) used for the diagnosis of OSCC patients vs healthy controls (**A**). The AUCs (areas under the ROC curves) are 0.708 for *P. gingivalis* IgG, 0.543 for *F. nucleatum* IgG, and 0.613 for serum IL-6. (**B**) Clinical performances of *P. gingivalis* IgG, *F. nucleatum* IgG, and serum IL-6 as a diagnostic marker for discrimination of OSCC and non-OSCC controls in terms of accuracy, sensitivity, specificity, false negative rate (FNR), false positive rate (FPR).

To clarify the diagnostic potential of the tested serum values, receiver operating characteristic (ROC) curves were plotted to distinguish the 62 patients with OSCC from the 46 non-OSCC controls. As shown in Figure 2a, the AUCs (areas under the ROC curves) were 0.708 for *P. gingivalis* IgG, 0.543 for *F. nucleatum* IgG, and 0.613 for serum IL-6, with optimal cutoff values of 1.732, 1.492, and 175.863, respectively. When the cutoff values were applied, the specificity for *P. gingivalis* IgG was higher (84.4%) than that for serum IL-6 (71.1%), but the sensitivity was slightly lower (53.2% vs 59.7%). Figure 2b shows the diagnostic performance of each factor in terms of accuracy, sensitivity, specificity, the false negative rate (FNR), and the false positive rate (FPR), indicating good diagnostic accuracy based on serum *P. gingivalis* IgG and IL-6.

**Figure 3:**
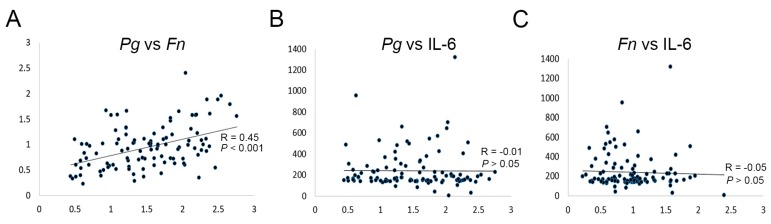
Correlation between serum IgG antibodies against *P. gingivalis* (**A**,**B**) and *F. nucleatum* (**A**,**C**) and IL-6 (**B**,**C**) in both OSCC patients and healthy controls.

The correlations between the serum levels of *P. gingivalis* and *F. nucleatum* IgG and IL-6 were determined. The titers of antibodies against *P. gingivalis* and *F. nucleatum* were strongly correlated, whereas associations between the titers of antibodies against periodontal pathogens and serum IL-6 levels were not observed (Figure 3).

### 2.2. Serum Interleukin-6 Is Associated with the 5-Year Survival Rate in OSCC Patients

To identify factors that could predict the prognosis of OSCC patients, first, the association between the clinicopathological features of OSCC and the length of the survival period was evaluated and determined by multivariate analysis and the Mann-Whitney U test. Clinicopathological factors, including tumor size, had little value in predicting prognosis (data not shown). The analysis revealed that OSCC patients with negative lymph node metastasis showed a better prognosis than OSCC patients with positive lymph node metastasis (57.04 months vs. 50.37 months), although this difference was statistically insignificant.

**Figure 4:**
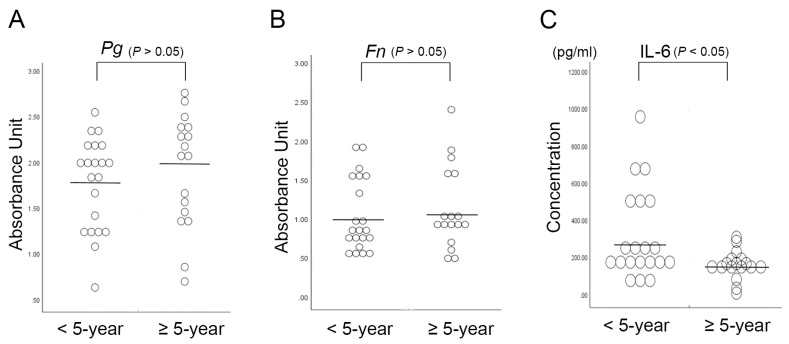
Serum *P. gingivalis* IgG and *F. nucleatum* values and serum IL-6 values in OSCC patients were analyzed according to the 5-year survival rate. The serum levels in OSCC patients who survived less than 5 years (*n* = 21) and more than 5 years (*n* = 17) were (**A**) *P. gingivalis* IgG, 1.77 ± 0.50 and 1.91 ± 0.62, (**B**) *F. nucleatum* IgG, 1.04 ± 0.47 and 1.13 ± 0.53, (**C**) IL-6, 359.40 ± 323.11 (pg/mL) and 158.48 ± 75.46 (pg/mL), respectively.

Subsequently, the association between the survival rate of patients and the serum levels of *P. gingivalis* and *F. nucleatum* IgG and IL-6 was analyzed using a Mann-Whitney U test and multivariate analysis. When evaluating the prognosis of cancer patients, the 5-year disease-free survival is the general standard; therefore, 24 surviving patients with less than 5 years of follow-up were excluded from the statistical analysis. Figure 4 shows a comparison of the serum values of antibodies against periodontal pathogens and the levels of IL-6 in 17 patients who survived for more than 5 years at the last clinical follow-up and 21 patients who passed away from OSCC before the 5-year period.

According to the analysis of the 5-year survival rate, OSCC patients with lower serum levels of Il-6 had a better survival rate, whereas the serum levels of *P. gingivalis* or *F. nucleatum* IgG were not correlated with the survival rate. Interestingly, significant value in predicting the prognosis of OSCC patients was observed for the serum value of IL-6, which showed significantly different values in OSCC patients who survived for more than 5 years than in those who survived for less than 5 years (158.5 ± 75.5 pg/mL and 359.4 ± 323.11 pg/mL, respectively).

Our study shows that high serum levels of the inflammatory cytokine IL-6 and *P. gingivalis* IgG are strongly correlated with OSCC. In addition, the serum IL-6 values were significantly correlated with the survival of OSCC patients, suggesting that these factors might serve as diagnostic biomarkers.

## 3. Discussion

Less extensive surgical treatment, which is a result of early diagnosis, can be a key determinant for improving long-term survival as well as cosmetic and functional outcomes in OSCC patients. Due to the seriousness of these needs, the identification of serum OSCC biomarkers that can be detected early and/or predict prognosis has been eagerly anticipated for many years [29,30,31]. Conventional serum markers are mostly oncoproteins that are produced during carcinogenesis, and markers such as carcinoembryonic antigen (CEA), squamous cell carcinoma antigen (SCCA), and carbohydrate antigen (CA) are well known. However, these markers are not applicable to the diagnosis of OSCC due to a lack of sensitivity and specificity.

Our previous studies demonstrated that *P. gingivalis* contributes to the increased aggressiveness of oral cancer by promoting epithelial-mesenchymal transition (EMT) and accelerates the invasion of cells by activating IL-8/MMPs [32]. Inaba et al. also reported that *P. gingivalis* promotes the invasion of OSCC cells by activating the ERK1/2-Ets1, p38/HSP27, and PAR2/NF-κB pathways and by inducing pro-MMP9 expression [33]. These findings suggest that a novel mechanism of progression and metastasis is involved in OSCC associated with periodontitis and that factors that are directly related to periodontitis, such as periodontal pathogens and related inflammatory cytokines, may serve as possible OSCC markers. However, the correlation between exposure to chronic periodontitis and oral cancer has not been clinically studied well, and few clinical studies have investigated the usefulness of serum levels of antibodies against periodontal pathogens in diagnosing OSCC, although chronic periodontitis is the disease most closely associated with the oral cavity. Studies on the diagnostic availability of antibodies against *P. gingivalis*, which is a major pathogen involved in chronic periodontitis, have been performed for cancers other than OSCC. In a cohort study of pancreatic cancer, high levels of antibodies to *P. gingivalis* were correlated with the risk of pancreatic cancer [34]. Another study reported on the diagnostic value of serum *P. gingivalis* IgG and IgA in esophageal squamous cell carcinoma (ESCC). This study is the first to report on the diagnostic value of *P. gingivalis* IgG in OSCC, and the diagnostic performance of a single IgG for OSCC in the present study was superior to that of IgG or IgA for ESCC and achieved a sensitivity of 53.2% and a specificity of 84.4%. The study on ESCC showed the diagnostic usefulness of *P. gingivalis* serum antibodies, as evidenced by high sensitivity and specificity of 68.75% and 68.46%, respectively, that were achieved, but this effect was due to the combination of *P. gingivalis* IgG and IgA [35]. In addition, the usefulness of serum antibodies against *P. gingivalis* in predicting the prognosis of OSCC was not clear in this study, in contrast to the positive association between the *P. gingivalis* antibody titer and prognosis that was found for other types of cancers. Studies of ESCC and orodigestive cancers have shown that a higher serum level of *P. gingivalis* IgG is associated with a worse prognosis than a lower serum level. However, the prognosis for ESCC was determined on the basis of the 3-year survival rate and the optimal cutoff value of the *P. gingivalis* IgG titer, whereas our study analyzed the correlation based on 5-year disease-free survival and compared the length of the survival period and the actual value of the level of *P. gingivalis* IgG. In addition, the study that observed higher mortality for patients with orodigestive cancers with high serum *P. gingivalis* IgG was mostly limited to colorectal and pancreatic cancers, and only 4 cases of lip, oral, and pharyngeal cancer deaths were included in the analysis [35,36]. Thus, the discrepancy for the usefulness of the *P. gingivalis* antibody titer as a prognostic indicator may be due to differences in cancer types, the criteria used for the survival rate, and the analysis method. In this study, *P. gingivalis* IgG antibody has been recognized as an important marker for diagnosing OSCC and predicting prognosis of patients, but the marker has also been identified to be correlated with ESCC and pancreatic cancer. For the development of diagnostic markers with higher specificity, a modified application of *P. gingivalis* IgG combined with other markers may be used, in addition to identifying new markers that can be used to specifically detect OSCC. In this study, we investigated the correlation between the serum levels of antibodies against *F. nucleatum*, which is another well-known periodontal pathogen, and OSCC. However, the results for *F. nucleatum* did not demonstrate any diagnostic value in our study. Through further studies of the serum values of antibodies against periodontal pathogens as well as various inflammatory mediators, more specific diagnostic markers could be developed.

IL-6 is implicated in various cancers in suppressing apoptosis and accelerating uncontrolled cell growth via activating growth factor and related signaling pathways [37]. Although the role of IL-6 in cancer development and progression is clear, studies on the serum levels of IL-6 in healthy controls and OSCC patients have shown contradictory results. The data showed no differences in the serum IL-6 levels in OSCC patients compared with those in healthy controls [38]. In contrast, two recent studies strongly suggested a positive correlation between IL-6 and cancer development by observing much higher levels of serum IL-6 in oral cancer patients than in healthy controls [29,39]. These contradictory results may be due to differences in the sensitivity of the assays used. The study that reported a negative role of serum IL-6 in diagnosing OSCC found serum IL-6 values of approximately 3–4 pg/mL in both the healthy control and OSCC groups. Compared to the low detection level for serum IL-6, two later studies observed much higher serum IL-6 values of approximately 10 pg/mL and 993 pg/mL, respectively, in controls and OSCC patients. Our results also revealed a high serum IL-6 value (average value: 359.40 pg/mL) in OSCC patients. In our study, we suggest that IL-6 is not only a good biomarker in terms of diagnostic accuracy (sensitivity and specificity of 59.7% and 71.1%, respectively) but also a good prognostic factor with high predictability (p < 0.029) for OSCC. The study by Schiegnitz E indicated that IL-6 is an independent prognostic factor of overall survival by demonstrating increased survival after 18 months in OSCC patients with a low serum IL-6 value [29]. This finding is supported by the present study, which observed lower serum IL-6 values in patients with increased 5-year cancer-free survival. In addition, it is worth considering that when determining the prognosis of cancer, 5-year survival is regarded as cancer-free survival. Therefore, our study further confirms the usefulness of the serum IL-6 value as a prognostic indicator. Though both the serum level of *P. gingivalis* IgG and serum IL-6 were increased in OSCC patients, no correlation between those two markers was observed. This finding implies that they are independent parameters and may be implicated in different stages or pathogenic mechanisms during oral cancer progression. In addition, *P. gingivalis* IgG showed a low false negative rate and a high false positive rate, while IL-6 exhibited an inverse tendency. A combination of these two independent markers may decrease their limit of detection and may be superior to using a single marker, but this must be confirmed in a future study.

In summary, the correlation between chronic inflammation and cancer has been extensively studied and proven for numerous types of cancer [12,13,40]. However, the roles of chronic inflammation and periodontitis in oral cancer progression have not been actively investigated, a few experimental studies using *P. gingivalis* and epidemiological studies have suggested a correlation [21,22,23]. Through the observation of a significant increase in the titers of serum antibodies against *P. gingivalis* as well as serum IL-6 in OSCC patient samples in the present study, the role of periodontitis and/or *P. gingivalis* in oral cancer progression has been clinically indicated. In addition, the present study suggests the possibility of using both serum IL-6 and *P. gingivalis* IgG as diagnostic and/or prognostic markers, though the specificity and sensitivity of the markers need to be improved. Further study should be focused on increasing the diagnostic potential by using a combination of several individual biomarkers. Diagnostic markers for oral cancer with increased predictive accuracy could be developed from further studies that investigate various combinations of chronic periodontitis-related factors, such as the titers of antibodies against periodontal pathogens and pro-inflammatory cytokines.

## 4. Materials and Methods

### 4.1. Study Subjects

Serum samples from 62OSCC patients who underwent surgery at the Department of Otorhinolaryngology of Pusan National University Hospital were obtained before surgery. The clinical stage of OSCC was classified in accordance with the eighth edition of the American Joint Committee on Cancer (AJCC). In addition, 46 healthy volunteers without evidence of comorbid disease who visited the Department of Oral and Maxillofacial Surgery in the hospital for oral care were recruited as healthy controls. Serum samples were stored at −80 °C until use, and all measurements were made in triplicate. All patients provided informed consent before joining this study. Ethical permission for this study was granted by the Institutional Review Board (IRB) of Pusan National University Dental Hospital (IRB No. PNUDH-2017-009; 29-March-2017).

### 4.2. Enzyme-Linked Immunosorbent Assay

*P. gingivalis* strain 381 and *F. nucleatum*, which were used as antigens in our experiment, were cultured anaerobically and grown in GAM broth (Nissui, Tokyo, Japan) at 37 °C. The levels of antibodies specific for *P. gingivalis* and *F. nucleatum* in the serum specimens were determined by a modified ELISA. The bacterial cells were grown to an optical density of 0.1 at 660 nm and extracted. The *P. gingivalis* or *F. nucleatum* antigens were coated onto 96-well assay plates (Corning, Cambridge, MA, USA) using 100 ng/well of sonicated extracts. Diluted serum samples (1:50) were applied to the wells precoated with antigen as described above and were incubated at 4 °C for 16 h. Bound human IgG was detected with an alkaline phosphatase-conjugated anti-human IgG (Jackson ImmunoResearch, West Grove, PA) followed by development with o-phenylenediamine (OPD) (Sigma, St. Louis, MO, USA). The reproducibility of the ELISAs was validated by testing and analyzing the intra- and interplate differences. The antibody levels were expressed as absorbance units.

The Il-6 serum levels were examined using a highly specific quantitative sandwich ELISA kit (Invitrogen, Carlsbad, CA, USA) according to the manufacturer’s instructions. The optical density was measured at 450 nm using a microplate reader. The concentrations of IL-6 were calculated on the basis of a standard curve.

### 4.3. Statistical Analysis

The statistical analyses were performed using the SPSS v23 software package (SPSS, Chicago, IL, USA). The data are expressed as the mean ± standard deviation (SD), and the frequencies and the proportions of the available relevant variables in the study population were described. To assess the relationship between the serum markers and the 5-year survival rate, we restricted the analysis to individuals who were monitored for more than 5 years (*n* = 38). The comparisons between the groups were performed by testing the mean differences, multivariate analysis, repeated ANOVA, or a Mann-Whitney U test. The significance of the prognostic factors for survival was analyzed with a Mann-Whitney U test. *p* values <0.05 were considered “statistically significant.”

## 5. Conclusions

This is the first study to demonstrate the usefulness of the levels of serum antibodies against periodontal pathogens as biomarkers for oral cancer. In addition, the remarkable increase in IL-6, which is a representative inflammatory cytokine, in the serum of patients with OSCC compared to that in normal controls provides additional evidence that inflammation is implicated in the pathogenesis of OSCC. A higher level of serum IL-6 in OSCC patients was also highly correlated with a worse prognosis, indicating the possibility of the utilization of the serum IL-6 level as a prognostic factor.

## Figures and Tables

**Table 1:** Association between serum IgG antibodies against *P. gingivalis* and the clinicopathological features of OSCC.

Variables		lgG Titer (Mean ± SD)	*p*
**Age**	≤60 (24 (38.7%))	1.61 ± 0.51	0.34
	>60 (38 (61.3%))	1.74 ± 0.60	
**Gender**	males (45 (72.6%))	1.70 ± 0.54	0.77
	Females (17 (27.4%))	1.65 ± 0.64	
**Tobacco Use**	No (39 (65.0%))	1.67 ± 0.58	0.74
	Yes (21 (35.0%))	1.73 ± 0.59	
**Alcohol Use**	No (37 (59.7%))	1.70 ± 0.61	0.70
	Yes (25 (40.3%))	1.68 ± 0.52	
**Histopathologic Grade**	I (21 (35.6%))	1.78 ± 0.44	0.16
	II (26 (44.1%))	1.53 ± 0.59	
	III (6 (10.2%))	1.55 ± 0.73	
	IV (6 (10.2%))	2.03 ± 0.39	
**Lymph Node Metastasis**	No (33 (59.7%))	1.63 ± 0.63	0.38
	Yes (29 (40.3%))	1.76 ± 0.49	
**Tumor Size**	T1 (25 (42.4%))	1.62 ± 0.58	0.74
	T2 (16 (27.1%))	1.61 ± 0.44	
	T3 (5 (8.5%))	1.89 ± 0.67	
	T4 (13 (22.0))	1.74 ± 0.68	
**TNM Stage**	I (19 (30.6%))	1.59 ± 0.63	0.53
	II (9 (14.5%))	1.54 ± 0.52	
	III (11 (17.2%))	1.74 ± 0.45	
	IV (23 (37.1%))	1.81 ± 0.59	

**Table 2:** Association between serum IgG antibodies against *F. nucleatum* and the clinicopathological features of OSCC.

Variables		lgG Titer (Mean ± SD)	*p*
**Age**	≤60 (24 (38.7%))	1.00 ± 0.52	0.78
	>60 (38 (61.3%))	0.99 ± 0.43	
**Gender**	males (45 (72.6%))	1.00 ± 0.42	0.45
	Females (17 (27.4%))	0.99 ± 0.58	
**Tobacco Use**	No (39 (65.0%))	1.03 ± 0.49	0.76
	Yes (21 (35.0%))	0.96 ± 0.44	
**Alcohol Use**	No (37 (59.7%))	0.96 ± 0.45	0.33
	Yes (25 (40.3%))	1.05 ± 0.49	
**Histopathologic Grade**	I (21 (35.6%))	1.04 ± 0.50	0.64
	II (26 (44.1%))	0.91 ± 0.43	
	III (6 (10.2%))	1.05 ± 0.52	
	IV (6 (10.2%))	1.11 ± 0.45	
**Lymph Node Metastasis**	No (33 (59.7%))	1.02 ± 0.49	0.99
	Yes (29 (40.3%))	0.98 ± 0.45	
**Tumor Size**	T1 (25 (42.4%))	1.04 ± 0.50	0.69
	T2 (16 (27.1%))	0.92 ± 0.48	
	T3 (5 (8.5%))	0.98 ± 0.58	
	T4 (13 (22.0))	1.06 ± 0.42	
**TNM Stage**	I (19 (30.6%))	1.08 ± 0.54	0.12
	II (9 (14.5%))	0.87 ± 0.46	
	III (11 (17.2%))	0.77 ± 0.35	
	IV (23 (37.1%))	1.09 ± 0.43	

**Table 3:** Association between the serum levels of IL-6 and the clinicopathological features of OSCC.

Variables		IL-6 (pg/mL) (Mean ± SD)	*p*
**Age**	≤60 (24 (38.7%))	303.9 ± 230.9	0.30
	>60 (38 (61.3%))	256.6 ± 228.2	
**Gender**	males (45 (72.6%))	260.2 ± 170.3	0.94
	Females (17 (27.4%))	314.0 ± 342.3	
**Tobacco Use**	No (39 (65.0%))	297.4 ± 262.5	0.38
	Yes (21 (35.0%))	229.7 ± 147.2	
**Alcohol Use**	No (37 (59.7%))	304.9 ± 265.3	0.23
	Yes (25 (40.3%))	230.6 ± 154.1	
**Histopathologic Grade**	I (21 (35.6%))	277.5 ± 288.3	0.59
	II (26 (44.1%))	266.4 ± 200.5	
	III (6 (10.2%))	315.5 ± 189.8	
	IV (6 (10.2%))	317.3 ± 236.4	
**Lymph Node Metastasis**	No (33 (59.7%))	291.1 ± 273.7	0.92
	Yes (29 (40.3%))	256.5 ± 165.9	
**Tumor Size**	T1 (25 (42.4%))	237.9 ± 144.4	0.85
	T2 (16 (27.1%))	338.3 ± 330.2	
	T3 (5 (8.5%))	226.0 ± 145.1	
	T4 (13 (22.0))	293.1 ± 258.5	
**TNM Stage**	I (19 (30.6%))	232.9 ± 150.7	0.82
	II (9 (14.5%))	396.8 ± 404.9	
	III (11 (17.2%))	239.1 ± 180.5	
	IV (23 (37.1%))	279.1 ± 208.8	

## Abbreviations

OSCC	Oral Squamous Cell Carcinoma
ESCC	Esophageal Squamous Cell Carcinoma
IL-6	Interleukin-6
ROC	Receiver Operating Characteristic
AUC	Area Under the ROC Curve
